# Microwave-Based State Diagnosis of Three-Way Catalysts: Impact Factors and Application Recommendations

**DOI:** 10.3390/s24134091

**Published:** 2024-06-24

**Authors:** Carsten Steiner, Vladimir Malashchuk, David Kubinski, Gunter Hagen, Ralf Moos

**Affiliations:** 1 Bayreuth Engine Research Center (BERC), Department of Functional Materials, University of Bayreuth, 95447 Bayreuth, Germany; 2 Ford Motor Company, Dearborn, MI 48124, USA; dkubinsk@ford.com

**Keywords:** ceria, oxygen storage capacity (OSC), three-way catalyst (TWC), exhaust gas aftertreatment, microwave cavity perturbation, gasoline engine, on-board diagnosis (OBD)

## Abstract

This study reassesses an overview of the potential of the radio frequency (RF)-based state diagnostics of three-way catalysts (TWC) based on a previous study with an emphasis on the defect chemistry of the catalyst material during reoxidation and reduction. Some data are based on the previous works but are newly processed, and the signal parameters resonant frequency and inverse quality factor are evaluated with respect to applicability. The RF-based method uses electromagnetic resonances in a cavity resonator to provide information on the storage level of the oxygen storage component. The analysis focuses on a holistic investigation and evaluation of the major effects influencing the RF signal during operation. On the one hand, the response to the oxygen storage behavior and the resolution of the measurement method are considered. Therefore, this study merges original data from multiple former publications to provide a comprehensive insight into important measurement effects and their defect chemistry background. On the other hand, the most important cross-sensitivities are discussed and their impact during operation is evaluated. Additionally, the effect of catalyst aging is analyzed. The effects are presented separately for the two resonant parameters: resonant frequency and (unloaded) quality factor. Overall, the data suggest that the quality factor has a way higher signal quality at low temperatures (<400 °C) and the resonant frequency is primarily suitable for high operating temperatures. At most operating points, the quality factor is even more robust against interferences such as exhaust gas stoichiometry and water content. Correctly estimating the catalyst temperature is the most important factor for reliable results, which can be achieved by combining the information of both resonant signals. In the end, the data indicate that microwave-based state diagnosis is a powerful system for evaluating the oxygen storage level over the entire operating range of a TWC. As a research tool and in its application, the system can therefore contribute to the improvement of the emission control of future gasoline vehicles.

## 1. Introduction

The three-way catalyst (TWC) plays a crucial role in reducing harmful emissions from internal combustion engines, particularly in vehicles. Its primary function is associated with the catalytic conversion of the major pollutants nitrogen oxides (NO*_x_*), carbon monoxide (CO), and hydrocarbons (HC) [1,2,3,4,5]. The widespread use of three-way catalysts has been instrumental in reducing air pollution and addressing the adverse effects of automotive emissions on human health and the environment. As a result, TWCs have become a standard feature in the exhaust systems of conventional gasoline and also hybrid vehicles globally [6,7,8,9,10]. The ongoing trend towards stricter emission standards worldwide requires continuous improvement of three-way catalysts and their control. Reducing emissions at low temperatures is still a key issue [10,11]. Furthermore, there is still interest in increasing catalyst durability [12,13,14,15,16,17].

One of the key components of a TWC is the oxygen storage component made of ceria-zirconia (CZO), which is designed to buffer fluctuations in the oxygen stoichiometry of the exhaust gas. During oxygen-rich conditions, i.e., lean-burn operation, the mixed oxide stores excess oxygen by oxidizing Ce^3+^ to Ce^4+^. Under rich conditions, the reverse reaction promotes the reduction of CZO. For the technical application, it is essential to control the storage level *θ*_TWC_ of the oxygen storage component. To effectively reduce raw emissions during operation, the fully oxidized and reduced states must be avoided, as they are associated with emission breakthroughs [18].

The current oxygen storage level *θ*_TWC_ in today’s vehicles is generally only estimated from the stoichiometric balance of two oxygen sensors upstream and downstream of the TWC (and kinetic models) [2,3,19,20,21]. This method only allows for an indirect estimation of the oxygen storage level *θ*_TWC_. To overcome this disadvantage, the radio frequency-based (RF) state diagnosis was introduced a couple of years ago, and it can directly determine the oxygen storage condition during operation [22,23]. This approach is based on the principles of microwave cavity perturbation (MCP)—a method widely used for dielectric material characterization [24]. In this case, the housing of the TWC is used as a cavity resonator. Electromagnetic energy (microwaves in the GHz range) is coupled into the cavity via coupling elements. At discrete frequencies, standing waves are excited, known as electromagnetic resonances. The resonant characteristics are in turn related to the dielectric properties of the filling medium in the resonator, including the TWC [25,26]. The dielectric response of CZO, in turn, depends on the oxidation state (or oxygen non-stoichiometry) of the mixed oxide, which was shown in recent microwave studies on both CZO and ceria [26,27,28,29]. Higher non-stoichiometries massively activate the dielectric losses due to the increase in conductivity (factor > 1000) by the small polaron (SP) hopping mechanism in the material [30,31,32,33]. The polarization in CZO is also increased by the chemical reduction (factor ≈ 2) [27,28,34].

Studies on the state diagnosis of TWCs have covered various topics in the past. In particular, the fundamental properties of the RF signal with respect to oxidation state have been investigated several times [25,35,36,37,38,39]. Furthermore, the RF system offers advantages over the classic oxygen sensors, as the point of maximum conversion can be determined more precisely [40]. The effect of catalyst aging was also considered and initial approaches were presented to evaluate the aging state with the RF system [25].

However, older studies exclusively focus on the resonant frequency *f*_res_, which provides information about the polarization of the oxygen storage component. Such studies also used different setups and catalysts, which also makes it difficult to cross-compare data. Interestingly, a recent investigation suggests that the quality factor *Q*_0_ as a second resonant signal is more sensitive to determining changes in the oxidation state of the TWC, particularly at low temperatures [35]. Instead of permittivity, the effect on *Q*_0_ is primarily based on changes in the attenuation of a resonance, which is linked to the dielectric loss of CZO. However, analyses of cross-sensitivities and their classification for engine operation (also in comparison to the resonant frequency) have been lacking to date. This is precisely where the present work ties in to give a more holistic assessment of the RF system for both resonant parameters (resonant frequency and inverse quality factor). Therefore, one focus of this study is to analyze the advantages of both resonant parameters. For this purpose, the temperature ranges close to catalyst light-off to higher temperatures up to 600 °C will be investigated. In addition, the signal quality of both resonant signals was evaluated in terms of their signal-to-noise ratio (SNR) and as a function of temperature and oxidation level. As the dielectric properties of CeO_2_ and CZO [27,28] have been investigated in recent microwave studies, these results are used to categorize and interpret the TWC results in light of defect chemistry. This will be the second focus of the present study.

## 2. Fundamentals, Materials and Methods

**Figure 1:**
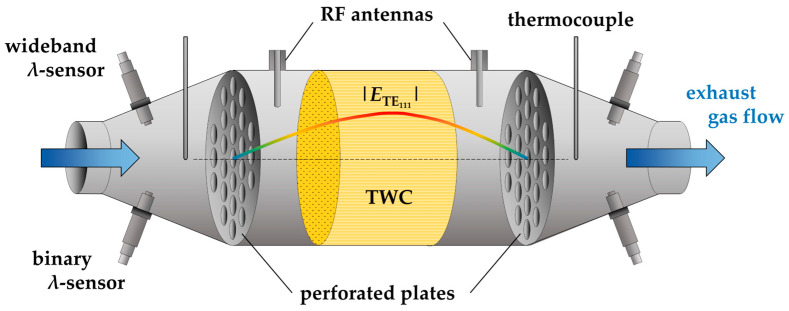
Schematic illustration of a typical setup for the RF-based state diagnosis of three-way catalytic converters, with the electrical field distribution of the TE_111_ mode along the symmetry axis of the cavity resonator.

The analysis is partly based on data previously published in [28,35], which has been processed in a different manner for this study. In addition, new results are shown using the same measurement setup. The configuration shown in Figure 1 is therefore only briefly described here. Further details can be found in [35]. The state diagnosis setup for the three-way catalyst consists of the housing, the coupling elements, which are often labeled as antennas, and the sensor systems for exhaust gas analysis in the cones up- and downstream of the catalytic converter. For the latter, a binary lambda probe (LSF 4.2, BOSCH, Gerlingen, Germany), a wideband lambda probe (LSF 4.9, BOSCH, Gerlingen, Germany), and a thermocouple (TC) are used in each case. The TC tips are located in the center of the canning cross section and the exhaust temperature is calculated from the arithmetic mean of both sensors. In addition, the gas concentrations downstream of the catalyst are measured by a FTIR gas analyzer (Multigas 2030, MKS Instruments, Andover, MA, USA).

The cavity resonator is represented by the catalyst canning. Its cylindrical geometry is formed by perforated steel plates on the up- and downstream sides with the TWC positioned in the center of the canning. The transmitted RF power is measured via two antennas using a vector network analyzer (VNA, ShockLine MS46322A, Anritsu, Atsugi, Japan). The results shown here use the TE_111_ resonant mode to determine the dielectric properties of the TWC. Its field distribution along the symmetry axis of the resonator is depicted in Figure 1. The TWC is therefore centered in the maximum of the electric field. For this study, two setups with different geometries are used. A large Ø4.66″ variant (Setup A) with low gas velocities for more precise analyses (gas hourly space velocity *GHSV* = 1000 h^−1^) and a smaller Ø1.66″ version to simulate dynamic operating conditions (*GHSV* = 32000 h^−1^). To analyze the oxygen storage behavior of both TWCs, the three-way catalysts were alternatingly reduced and re-oxidized using synthetic rich and lean gas mixtures provided by a laboratory test bench. For details on the gas composition, we again refer to [35]. Based on these experiments, the current oxygen storage level *θ*_TWC_ can be determined from the balance of the oxygen sensors [41]:

(1) $$\theta_{\mathrm{TWC}}=\;\frac{p_{0}M_{O_{2}}}{{\mathrm{RT}}_{0}}\frac{1+\frac{x}{4}}{1+\frac{x}{2}+\frac{1+\frac{x}{4}}{y_{O_{2}}}(1-y_{O_{2}})}\mathrm{GHSV}\int_{t_{1}}^{{\;t}_{2}}{|\lambda }_{\mathrm{up}}-\lambda_{\mathrm{down}}|dt,$$

with $p_{0}$ = 1.013 bar and $T_{0}$ = 273.15 K at standard conditions, the molar weight of oxygen $M_{O_{2}}$ = 32 u = 32⋅1.661⋅10^−27^ kg, the universal gas constant *R* = 8.314 J/(mol K), the signal of the UEGO sensors upstream (*λ*_up_) and downstream (*λ*_down_), as well as the molar H/C ratio *x* of the fuel and the oxygen fraction $y_{O_{2}}$ of the air, used for combustion.

The balance maxima are equivalent to the oxygen storage capacity (*OSC*) for rich-lean changes and the oxygen release capacity (*RSC*) in case of lean-rich changes. Both *OSC* and *RSC* are temperature-dependent [42,43,44]. In RF experiments, the oxygen storage level *θ*_TWC_ is evaluated either via the resonant frequency *f*_res_ or the inverse quality factor *Q*_0_^−1^. In general, their relative signal amplitudes are used to provide a better comparison of the resonant signal data:

(2) $${\Delta f}_{\mathrm{res},\mathrm{rel}}=\frac{{\Delta f}_{\mathrm{res}}}{f_{\mathrm{res},\mathrm{ref}}}=\frac{f_{\mathrm{res}}-{\;f}_{\mathrm{res},\mathrm{ref}}}{f_{\mathrm{res},\mathrm{ref}}},$$

(3) $${\Delta Q}_{0,\mathrm{rel}}{\;}^{-1}=\frac{{\Delta Q}_{0}{\;}^{-1}}{Q_{0,\mathrm{ref}}{\;}^{-1}}=\frac{Q_{0}{\;}^{-1}-Q_{0,\mathrm{ref}}{\;}^{-1}}{Q_{0,\mathrm{ref}}{\;}^{-1}}.\;$$

Throughout many experiments, the fully oxidized catalyst has proved to be a reliable reference, whose resonant signals are expressed by *f*_res,ref_ and *Q*_0,ref_^−1^ [25,35,36,39]. As mentioned before, the two resonant signal amplitudes (${\Delta f}_{\mathrm{res},\mathrm{rel}}\;\mathrm{and}\;{\Delta Q}_{0,\mathrm{rel}}{\;}^{-1}$) are based on different physical effects inside the resonator. On the one hand, the amplitude in the resonant frequency Δ*f*_res_ is a function of the change in the relative permittivity $\Delta \varepsilon_{r,\mathrm{TWC}}^{\prime }$ of the three-way catalyst. On the other hand, the amplitude of the inverse quality factor ${\Delta Q}_{0}{\;}^{-1}$ correlates with the change in the dielectric losses $\Delta \varepsilon_{r,\mathrm{TWC}}^{\prime \prime }$:

(4) $${\Delta f}_{\mathrm{res}}\;∼\Delta \varepsilon_{r,\mathrm{TWC}}^{\prime }=\varepsilon_{r,\mathrm{TWC}}^{\prime }-\varepsilon_{r,\mathrm{ref}}^{\prime }\;,$$

(5) $${\Delta Q}_{0}{\;}^{-1}\;∼\Delta \varepsilon_{r,\mathrm{TWC}}^{\prime \prime }=\varepsilon_{r,\mathrm{TWC}}^{\prime \prime }-\varepsilon_{r,\mathrm{ref}}^{\prime \prime }.\;$$

The changes of both dielectric properties $\Delta \varepsilon_{r,\mathrm{TWC}}^{\prime }$ and $\Delta \varepsilon_{r,\mathrm{TWC}}^{\prime \prime }$ are again referenced to the fully oxidized three-way catalyst, with its values $\varepsilon_{r,\mathrm{ref}}^{\prime }$ and $\varepsilon_{r,\mathrm{ref}}^{\prime \prime }$. Another important parameter for evaluating the sensoring properties of both resonant signals is their sensitivity to changes in the oxygen storage level *θ*_TWC_. In the case of the TWC, this sensitivity is actually a function of temperature and, to a lesser extent, also of *θ*_TWC_. For a simpler assessment, this study evaluates the (temperature-dependent) averaged sensitivity $\bar{S}$^RSC^ of each resonant signal in terms of the total *RSC* of the TWC, which is defined as follows:

(6) $${\bar{S}}^{\mathrm{RSC}}=\frac{{|S}_{\max}|}{\mathrm{RSC}}.\;$$

Here, *S*_max_ represents the maximum changes of the (relative) resonant signals (Δ*f*_res,rel_)_max_ or (Δ*Q*_0,rel_^−1^)_max_ when fully reducing the TWC during a lean-rich change (e.g., using the full *RSC* of the TWC). In order to assess the quality of a signal, the signal-to-noise ratio (*SNR*) of the signal should also be discussed, as it also provides information about the resolution of the signals:

(7) $$\mathrm{SNR}=\frac{{|S}_{\max}|}{\sigma_{S}}.\;$$

The *SNR* is the quotient of the maximum signal amplitude *S*_max_ of an RF signal and its standard deviation *σ*_s_. In general, the standard deviation is given by the following:

(8) $$\sigma_{S}=\frac{1}{N}\;\overset{N}{\underset{i=1}{\sum }}{{(x}_{i}-\bar{x})}^{2}\;.\;$$

*N* represents the number of data points, *x*_i_ represents the *i*-th measured value, and the mean value of the measured variable is expressed by $\bar{x}$. The standard deviation *σ*_s_ is a function of temperature and $\theta_{\mathrm{TWC}}$, and was therefore determined for both the fully oxidized and the reduced three-way catalyst for each resonant signal *f*_res_ and *Q*_0_^−1^.

Additionally, in this study, the correlation between the dielectric properties of the TWC and the defect chemistry of the CZO-based oxygen component will be addressed at key points. In this context, the most important defect chemical mechanisms of CZO and their importance for the TWC will be briefly described in the following section:

As mentioned before, the ability of cerium ions to exist in multiple oxidation states (Ce^3+^, Ce^4+^) is one of the key features contributing to the defect chemistry of CZO [33,45,46]. This redox behavior is associated with the formation of oxygen vacancies $V_{O}^{••}$, which are typically the major defects in the crystal lattice. In Kröger–Vink notation, their formation follows the equation as follows [46,47]:

(9) $${2\;\mathrm{Ce}}_{\mathrm{Ce}}^{x}{+4\;O}_{O}^{x}\;↔{\;2\;\mathrm{Ce}}_{\mathrm{Ce}}^{/}{+3\;O}_{O}^{x}{+V}_{O}^{••}+\frac{1}{2}{\;O}_{2}.$$

The ${\mathrm{Ce}}_{\mathrm{Ce}}^{x}$ and ${\mathrm{Ce}}_{\mathrm{Ce}}^{/}$ cations are equivalents to Ce^4+^ and Ce^3+^, respectively. The oxygen anions in the lattice are denoted by O^2–^. The electronic nature of ceria is mainly determined by the electrons localized to Ce^3+^. These electrons can migrate to adjacent Ce^4+^ via a small-polaron hopping mechanism (SP hopping) [30,48,49]. Therefore, the electronic conductivity *σ* in CZO is a function of the concentration of oxygen vacancies $V_{O}^{••}$. This behavior is also expressed by the electroneutrality condition, as follows [31]:

(10) $${2\;[V}_{O}^{••}]=n.$$

Here, *n* represents the concentration of charge carriers, specifically the concentration of reduced cerium cations [Ce^3+^]. The presence of zirconium ions further enhances the stability of these oxygen vacancies, creating a synergy in defect formation. The origin of this effect is attributed to the different sizes of the cerium and zirconium cations. The strain induced by this so-called sized effect acts as a driving force for the formation of oxygen vacancies in the lattice [50,51,52,53,54,55].

The activation of the accessible non-stoichiometry in CZO is highly dependent on the ratio of cerium to zirconium cations and affects the material’s oxygen storage capacity, which is an essential characteristic of TWC applications [14,56,57,58]. Along with the defect concentration, CZO also shows higher electronic conductivities compared to pure ceria [33,46,49,57,59]. As a result, ionic contributions, which are usually observed for pure ceria, play only a minor role in CZO. This applies even more for reduced CZO. Since this study also focuses on a differential analysis between the oxidized and reduced oxygen storage components, it is reasonable to assume that changes between these states are primarily due to changes in electronic conductivity. In this context, the correlation of the conductivity *σ* of a material and its dielectric losses $\varepsilon_{r}^{\prime \prime }$ is described by the following equation [24]:

(11) $$\varepsilon_{r}^{\prime \prime }=\varepsilon_{\mathrm{Pol}}^{\prime \prime }{+\sigma (\omega \varepsilon }_{0}{)}^{-1},$$

with the angular frequency *ω* of the electromagnetic wave and the electric field constant *ε*_0_ = 8.854⋅10^−12^ F/m. According to the MCP theory, the dielectric losses mainly affect the (inverse) unloaded quality factor *Q*_0_^−1^ of the resonance. This may be assumed to apply also to the RF setup for the state diagnosis. As shown in Equation (11), conductivity *σ* and polarization losses $\varepsilon_{\mathrm{Pol}}^{\prime \prime }$ both contribute to the total dielectric losses $\varepsilon_{r}^{\prime \prime }$. However, recent RF studies on CZO materials have indicated that the polarization losses may be negligible due to the high ohmic losses in CZO [27,28].

Studies have also shown that the relative permittivity $\varepsilon_{r}^{\prime }$ increases in reduced CZO. Generally, in ceramics, the ability of the lattice to align along an excitation field is closely related to its lattice vibrational modes (phonons) in the material [60]. A DFT study has shown that reducing CZO leads to increased excitation of existing and new vibrational modes in the lattice, which increases its dielectric response [34]. The effect also agrees quantitatively with experimental microwave data on CZO [28]. As mentioned above, these changes in the relative permittivity $\varepsilon_{r}^{\prime }$ primarily affect the resonant frequency *f*_res_ [24].

Besides dielectric properties, the defect chemistry of CZO also highly contributes to its catalytic performance, as CZO surfaces play a crucial role in facilitating redox reactions. The availability of oxygen vacancies promotes the adsorption and activation of reactant molecules [14,53,61]. Furthermore, the metal support interaction (MSI) between the high-surface-area support (CZO, Al_2_O_3_) and catalytically active metal nanoparticles (platinum, rhodium, and palladium) is a key mechanism for the outstanding results in the simultaneous conversion of NO*_x_*, CO, and HC of modern TWCs [62,63,64,65]. After outlining the experimental setups, methods, and scientific principles used in this work, the next section presents the results of the measurements.

## 3. Results and Discussion

### 3.1. RF-Based Monitoring of the Oxygen Storage Level

This study investigates the impact factors on the RF-based state diagnosis for the TWC and evaluates their effects on vehicle operation. The results will be discussed separately for the resonant frequency and the (inverse) quality factor. Initially, the response of the RF system to changes in oxygen storage is investigated. The investigation was conducted using Setup A within the temperature range of 280 to 550 °C. An initial study on the correlation of the resonant signals and the current oxygen storage level $\theta_{\mathrm{TWC}}$ has already been provided in [35]. This study goes beyond the previous data by providing more detailed information. Furthermore, subsequent findings regarding cross-sensitivities were also obtained at the same configuration, facilitating a direct comparison between the datasets.

**Figure 2:**
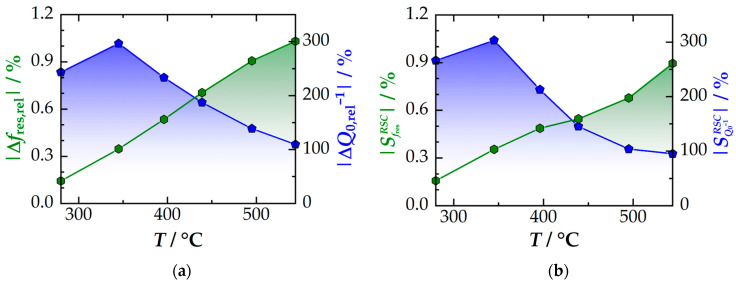
Overview of the temperature-dependent properties of both resonant signals *f*_res_ (green) and inverse quality factor *Q*_0_^−1^ (blue) with the absolute values of (**a**) the signal amplitudes and (**b**) the sensitivities when utilizing the full *RSC* of the TWC (lean-rich change). Adapted original data from [35].

Firstly, this study focuses on the temperature-dependent maximal signal amplitudes and sensitivities of the resonant signals when the oxygen storage capacity (*OSC* or *RSC*) of the TWC is fully utilized. Both parameters are shown for *f*_res_ (green) and *Q*_0_^−1^ (blue) in Figure 2a,b. Different trends for the two resonant signals can be derived from the graphs: As Figure 2a shows, the amplitude of the resonant frequency increases almost linearly with temperature. The signal amplitude is approximately 1%. In comparison, the signal amplitude of the inverse quality factor exhibits a maximum at 300–400 °C, which subsequently decreases at higher temperatures. Additionally, the signal amplitudes of up to 300% exceed those of *f*_res_ by more than two decades.

**Figure 3:**
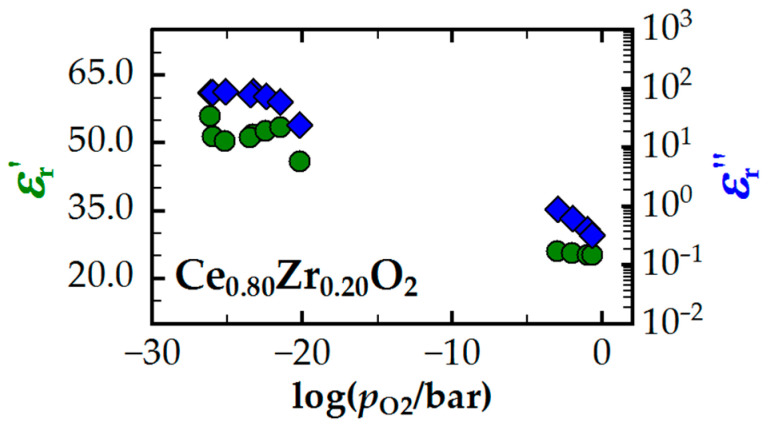
Relative dielectric permittivity $\varepsilon_{r}^{\prime }$ (green) and dielectric losses $\varepsilon_{r}^{\prime \prime }$ (blue) of a Ce_0.80_Zr_0.20_O_2_ powder sample as a function of *p*_O_2__, measured at approx. 1.2 GHz with the TE_010_ mode of a cylindrical cavity resonator at 600 °C. Adapted original data from [28].

The varying different orders of magnitude observed for the resonant signals can be explained by defect chemical effects. Beneficial information on this topic is provided by another study, which investigated the correlation between the defect chemistry of CZO and its dielectric properties in the microwave range [28]. Figure 3 displays excerpted results on the relative permittivity (green) and dielectric losses (blue) in the Ce_0.80_Zr_0.20_O_2_ powder sample. Such CZO compositions are representative of the oxygen storage material found in three-way catalysts [14] (even slightly different compositions do not significantly affect the fundamental conclusions that can be drawn from the results). The analysis shows that oxidized CZO (high oxygen partial pressures *p*_O_2__) has a dielectric permittivity $\varepsilon_{r}^{\prime }$ of approximately 25, which is approximately doubled by the chemical reduction at low *p*_O_2__. As explained in the previous section, changes in the dielectric permittivity primarily affect the resonant frequency and are based on the excitations of additional and existing phonon modes in the lattice [28,34]. In comparison, the dielectric loss $\varepsilon_{r}^{\prime \prime }$ in CZO increases significantly more during the reduction with more than two decades (note the logarithmic scale for $\varepsilon_{r}^{\prime \prime }$ in Figure 3). This effect correlates with the activation of the SP hopping mechanism and therefore with the concentration of oxygen vacancies [27,33,45]. In the microwave measurement, the dielectric loss is in turn linked to the inverse quality factor. Thus, the different magnitudes of the signal amplitudes of *f*_res_ and *Q*_0_^−1^ can be explained physically.

In addition, it is important to consider that in Figure 2 the relative signal amplitudes of both resonant signals are referenced to the oxidized full catalyst, e.g., the change in resonant signals of the RF-based state diagnosis depends on the catalyst geometry (monolith dimensions, cell density) and the catalyst materials, which includes not only the oxygen storage component, but also the substrate material, washcoat composition, and precious metals. In fact, the oxygen storage component represents only a minor part of the TWC. Therefore, the relative changes in the resonant signal are expected to be smaller than in microwave investigations on pure CZO. One can also deduce that signal amplitudes are affected by the operating temperature, as the dielectric properties of the full catalytic converter naturally change with temperature. In this context, different conclusions can be drawn for the impact of the two resonant signals:

In Figure 2a, it can be observed that the signal amplitude of *f*_res_ increases almost linearly with higher temperatures. This finding is consistent with earlier studies, which suggested that the signal amplitude of *f*_res_ correlates with the temperature-dependent oxygen storage capacity (*OSC*/*RSC*). Although this statement is not fundamentally wrong, it may not be entirely accurate. As shown in Figure 2b, the sensitivity of the measurement signal increases significantly with temperature, by almost a factor of 6 over the entire temperature range (280–550 °C). A minor part of this effect is due to referencing the oxidized full catalyst. Previous measurements have demonstrated that the resonant frequency of the oxidized catalyst decreases with temperature [25,35], resulting in a reduced reference value *f*_res,ref_. According to Equation (2), this effect increases both the (relative) signal amplitude and the sensitivity of the resonant frequency measurement. However, as the data of multiple studies show, the quantitative effect is in the range of a few percent (for example, see [35], where the *f*_res,ref_ changes from approximately 1.265 to 1.250 GHz over 280–550 °C). Therefore, the majority of the significant temperature activation of the amplitude of *f*_res_ must originate from another physical effect.

As previously mentioned, the resonant frequency is linked to the dielectric permittivity $\varepsilon_{r}^{\prime }$. The dielectric response of a metal oxide depends on the ability of its lattice to align itself along an electric excitation field. The mechanism is coupled to its lattice vibrational modes (optical phonons). Since DFT studies have shown that, in reduced CZO, a softening of both new and existing phonon modes is observed, this effect thereby increases its permittivity [34]. Furthermore, at higher temperatures, the atoms within the material have greater kinetic energy, resulting in an amplification of the vibrational modes. If, in reduced CZO, existing and new lattice vibrational modes are amplified (e.g., softened), then these phonon modes must commutatively be more activated by temperature (compared to oxidized CZO). Therefore, the polarization difference between reduced and oxidized CZO is expected to increase with higher temperatures. In the end, the same applies to the sensitivity, as well due to the growth of the physical measurement effect (aligning of the lattice). Both are consistently found in the experiments. Therefore, previous explanations that the resonant frequency signal correlates with the *OSC* are not entirely correct from a scientific perspective, as they imply a direct link between the signal amplitude and the defect concentration in CZO. Although the reduction is indeed the origin of the increased permittivity, this model alone does not explain the increasing sensitivity with higher temperatures. Instead, the physical relations suggest that besides *OSC*, the measuring effect itself increases with temperature.

When considering the inverse quality factor to determine the oxygen storage level some fundamental differences in the resonant frequency are observed. The maximum signal amplitude of *Q*_0_^−1^ (Figure 2a) and sensitivity (Figure 2b) are observed at low temperatures (300–400 °C). Beyond those temperatures, both parameters decrease significantly (by a factor of 3). This behavior suggests that beyond the light-off temperature, the quality factor amplitude initially increases with the higher *OSC* (linked to the defect concentration in CZO). The sensitivity remains almost constant within this operating window, and the high signal amplitudes (compared to the resonant frequency) are due to the massively higher conductivity in reduced CZO. Above 400 °C, the signal becomes increasingly decoupled from the further increase in *OSC* due to the significantly increasing total losses in the resonator. These losses originate from CZO but also from the other catalyst materials and reduce the quality factor to low levels. As a guide, even when using advanced fitting methods (as applied in this study) for MCP measurements in transmission mode, quality factors of more than 103 are usually recommended for precise dielectric characterization [66]. However, in the case of cavity resonators for state diagnosis, even in the oxidized state, values of only a few hundred are achieved. In the reduced state, the quality factor can easily drop below 50 (see, e.g., [35]). Since the relative signal amplitudes are also referenced to the oxidized state, both effects ultimately lead to a decreasing sensitivity at temperatures >400 °C.

Therefore, we can summarize that the resonant frequency and inverse quality factor respond differently to defect chemical processes in CZO (and therefore also the current level of instored oxygen). Additionally, the operating temperature significantly influences the underlying measurement effects. Based on this fundamental classification of the measurement signals, the advantages of the two resonant signals during operation will be investigated in more detail. The first step is to evaluate the signal-to-noise ratio (*SNR*), as the latter provides information about the resolution of the RF signals.

**Figure 4:**
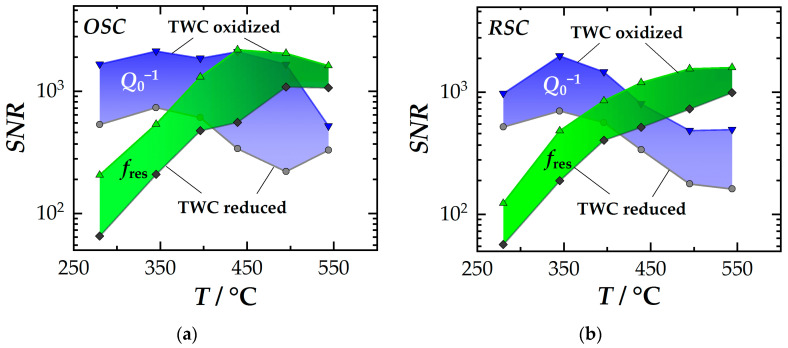
Signal-to-noise ratios (*SNR*) of the two resonant signals *f*_res_ (green) and *Q*_0_^−1^ (blue) over the temperature of the TWC, classified by catalyst state: (**a**) Data from the *RSC* balance (lean-rich *λ*-variations) and (**b**) the *OSC* balance (rich-lean variations). Adapted original data from [35].

The temperature-dependent *SNR* curves of *f*_res_ (green) and *Q*_0_^−1^ (blue) are shown in Figure 4a,b. Figure 4a contains the processed data from [35] for the *RSC* balance and Figure 4b for the *OSC* balance. As the data show, each resonant signal has an upper and a lower limit. The upper limit refers to the fully oxidized catalyst. Here, the quality factors are highest in the cavity resonator due to the small dielectric losses. The lower limit thus marks the completely reduced catalytic converter where quality factors are much lower. As another result, the standard deviations of both resonant signals generally increase with temperature and therefore have a negative impact on the *SNR*. In operation, the *SNR* lies between these two limits (the fully oxidized and reduced states should generally be avoided due to emission breakthroughs).

From the *SNR* data, again different trends for the resonant frequency and the inverse quality factor can be observed. The *SNR* of *f*_res_ increases significantly with temperature. However, at low temperatures (<400 °C), it is surpassed by the *SNR* of *Q*_0_^−1^ (note the logarithmic ordinate axis!). Regarding the quality factor, the *SNR* remains relatively constant up to a temperature of approximately 400 °C but decreases beyond this point. In contrast to *f*_res_, the *SNR* of the quality factor is slightly less dependent on temperature. For both RF signals, *SNR*s of around 10^3^ can be achieved in specific temperature windows. Thus, it is possible to realistically resolve relative changes in the oxygen storage level to within 1 ‰ through state diagnosis.

As a conclusion, *Q*_0_^−1^ is the recommended signal for low temperatures up to 400 °C. In this context, monitoring the catalyst light-off is crucial for reducing cold start emissions using appropriate control strategies. The data presented here clearly indicate that the quality factor is the more suitable signal for this purpose. For comparison, at 300 °C, the *SNR* of *f*_res_, averaging around 50, is more than one order of magnitude lower than that of *Q*_0_^−1^. The primary cause of this effect is the low signal amplitude of *f*_res_ under these conditions. Additionally, the evaluation by the *SNR* is noteworthy as it demonstrates that the *SNR* curves of both resonant signals intersect at higher temperatures. For higher temperatures (>450 °C), *f*_res_ is the more favorable RF signal. In contrast, this is the typical application for engine operation after the cold start.

### 3.2. Cross-Sensitivities and Interference Effects

**Figure 5:**
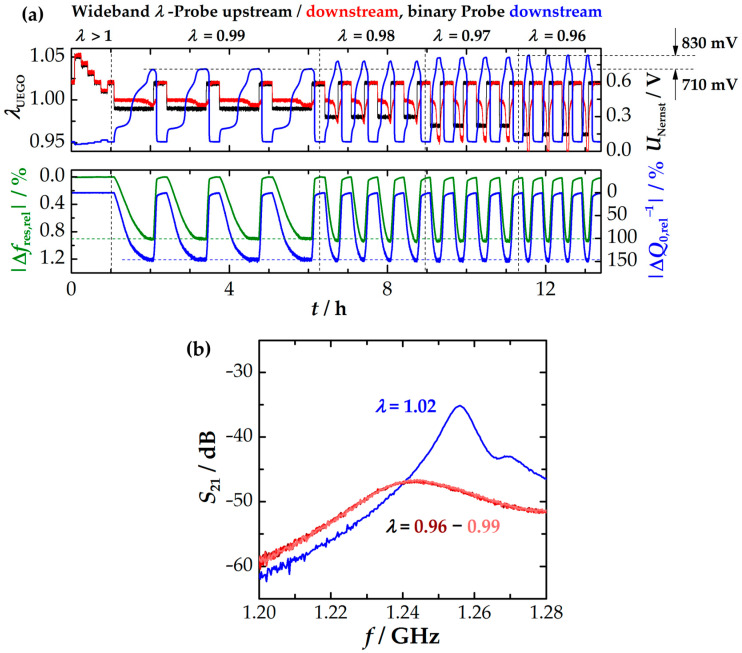
Experiment to determine the effect of oxygen stoichiometry at 500 °C with (**a**) upper plot: *λ* signals of the two wideband probes (upstream in black, downstream in red) and the Nernst voltage *U*_Nernst_ of the downstream binary probe (blue), bottom plot: RF signals *f*_res_ (green) and *Q*_0_^−1^ (blue) and (**b**) transmission spectra at different oxygen stoichiometries *λ*.

The following section will focus on evaluating cross-influences on the resonant signals. While the previous part of this work dealt more with the defect chemistry of the oxygen storage component and the understanding of the scientific background, the following sections will primarily discuss the interference effects from an application perspective to assess their significance for TWC control. Initially, the impact of the oxygen stoichiometry *λ* on the RF signal is investigated. This is because stoichiometry directly affects the *p*_O_2__ in the exhaust gas, which is again linked to the defect chemistry of CZO and therefore to its conductivity and dielectric properties (see Figure 3) [29,30]. The stoichiometry of the exhaust gas changes dynamically during engine operation, making a robust microwave signal particularly desirable. To achieve this, additional redox cycles (lean-rich and rich-lean switches) were conducted using Setup A at 500 °C with various exhaust gas stoichiometries in the range of 0.96 ≤ *λ* ≤ 1.05. The specific compositions of the synthetic exhaust gas mixtures are listed in Table 1. It is important to note that the stoichiometry was primarily determined by regulating the concentrations of O_2_, H_2_, and CO. The concentrations of CO_2_ and H_2_O remained constant. Figure 5a presents an overview of the experiment, displaying the signals from the wideband and binary probes in the upper plot and the two radio-frequency signals in the lower drawing. Additionally, the transmission spectra at different oxygen stoichiometries *λ* are presented in Figure 5b.

During the initial phase of the experiment (0 h ≤ *t* ≤ 1 h), the stoichiometry was varied only within lean compositions of 1.01 ≤ *λ* ≤ 1.05. The resulting change in *p*_O_2__ is indicated by step-wise changes in the Nernst voltage of the downstream binary *λ* probe (blue). As previously assumed, the RF signal remained largely constant under these conditions and was not affected by changes in the exhaust gas stoichiometry. Therefore, the oxidized catalyst state is a suitable reference for RF experiments on the oxygen storage level. Still, a change in the dielectric properties of CZO is expected from defect chemistry, because obviously both the relative permittivity $\varepsilon_{r}^{\prime }$ and dielectric losses $\varepsilon_{r}^{\prime \prime }$ in CZO increase with lower *p*_O_2__ in lean exhausts (see Figure 3). However, measurements have proved that changes in the dielectric response of CZO at high *p*_O_2__ are quantitatively small at high *p*_O_2__. For example, the dielectric loss in CZO is several decades smaller than at low *p*_O_2__ (see Figure 3 or [28]). Considering also the poor resonance qualities of the cavity resonator and the fact that the other materials of the catalytic converter, such as the substrate and support material, also contribute to and possibly even dominate the overall dielectric properties of the three-way catalytic converter, we can conclude that these (absolute) changes in the non-stoichiometry are way too small to significantly affect the RF signal.

During the second part of the experiment (*t* > 1 h), substoichiometric exhaust gas compositions were applied to the TWC ranging from 0.96 to 0.99. Re-oxidation occurred at *λ* = 1.02. Four cycles were run for each setting. The individual sections of the experiment are indicated by dashed vertical lines. The Nernst voltage of the downstream binary probe (blue) increased during the rich phase, indicating a decrease in *p*_O_2__ with lower lambda values. The change in *p*_O_2__ can be quantified by the rise in Nernst voltage from 710 to 830 mV (with a typical sensitivity of approximately 50 mV per *p*_O_2__ decade for low *p*_O_2__) to over two orders of magnitude. Here, the quality factor (blue) consistently shows a stable and constant value for the fully reduced oxygen storage (see Figure 5a, bottom plot). In contrast, the resonant frequency exhibits a slight shift towards lower values with higher oxygen deficits. In the range of 0.96 ≤ λ ≤ 0.99, the total change is less than 0.4 MHz. In contrast, the signal amplitude of *f*_res_ on the oxygen storage level of the catalyst (at 500 °C) is approximately 30 times greater (>11 MHz). Therefore, the relative deviation is ca. 3%, indicating that oxygen stoichiometry remains the primary impact factor of both RF signals. Indeed, the spectra under rich exhaust gas conditions are nearly identical, as shown in Figure 5b. However, it is worth noting that the quality factor is more resistant to changes in exhaust gas stoichiometry. Considering the CZO defect chemistry, this result appears plausible. As previous measurements have shown (e.g., see $\varepsilon_{r}^{\prime \prime }$ in Figure 3), the dielectric losses in CZO increase by several orders of magnitude during reduction (high → low *p*_O_2__), but do not change significantly at low *p*_O_2__ anymore. This effect is primarily due to the inhibition of the SP hopping mechanism at high Ce^3+^ concentrations [31,59]. For further elaboration, we refer to the microwave study of CZO in [28]. In contrast, the lower changes in polarization (maximum factor 2) could generally explain the higher interference effects on the resonant frequency.

**Figure 6:**
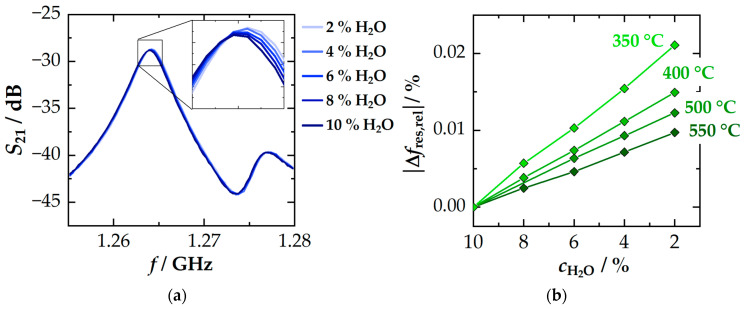
Effect of the H_2_O concentration on the RF signal: (**a**) transmission spectrum (*S*_21_) near the TE_111_ mode at 350 °C and (**b**) temperature-dependent effect on the relative change in resonant frequency Δ*f*_res,rel_.

Besides oxygen stoichiometry, the interaction of the TWC with water is also important to quantify for the RF-based diagnosis. The adsorption of water molecules on the nanostructured washcoat was observed to highly affect the resonance frequency during cold start. Here, the impact of adsorbed water was even suggested to be used for the determination of the catalyst aging state [25]. Instead, this study analyzes the impact in typical TWC operation windows at elevated temperatures (>300 °C) and will include results for the quality factor. Figure 5a shows the effect of different H_2_O concentrations on the transmitted power (*S*_21_) near the TE_111_ mode at 350 °C. As shown in the figure, an increase in water concentration leads to lower resonant frequencies (shift of the TE_111_ resonance peak) and a slightly higher attenuation of the maximum power (reduction of *S*_21_). The data also confirm that the effects on the resonant frequency are greater than those on the quality factor. In fact, it is difficult to quantify the effect on *Q*_0_^−1^, as it is within the resolution limits of the measurement system. As this is not the case for *f*_res_, the temperature-dependent impact on the (relative) change in resonant frequency Δ*f*_res,rel_ is shown in Figure 6b. The signal is referenced to *f*_res_ at *c*_H_2_O_ = 10% (typical operating conditions). The overall effect of a change in H_2_O concentration from 10% to 2% at 350 °C can be quantified as ≈ 0.021%. The effect on *f*_res_ obviously decreases with higher temperatures. This can be explained by a suppressed adsorption of water on the high surface area washcoat at higher temperatures, which reduces associated polarization mechanisms. The correlation between *f*_res_ and water concentration is virtually linear.

Considering the maximum signal amplitude of *f*_res_ to the oxygen storage level of the TWC at the same temperatures (Figure 2a), it is clear that, even at temperatures of 350 °C, the signal amplitude responds more strongly to changes in oxygen storage level by a factor of about 15. At 550 °C, this ratio is already close to 100. It can therefore be concluded that the water concentration can have a significant impact on the evaluation of the oxygen storage level exclusively for *f*_res_ at low temperatures (<350 °C). At higher temperatures, however, the effect of water can be neglected. For the quality factor, changes can hardly be resolved anyway. Even at 350 °C, deviations caused by *c*_H_2_O_ are estimated to be less than 1% of the quality factor’s signal amplitude to the *RSC*. Therefore, the quality factor can be considered independent of *c*_H_2_O_ over the entire temperature range, which again emphasizes the advantage of the quality factor at low temperatures. At this point, it is worth mentioning that a similar experiment was also carried out with the CO_2_ concentration. Here, no significant impact was detected. Both signals, *f*_res_ and *Q*_0_^−1^, are basically independent of the CO_2_ concentration.

### 3.3. Impact and Evaluation of Catalyst Aging

**Figure 7:**
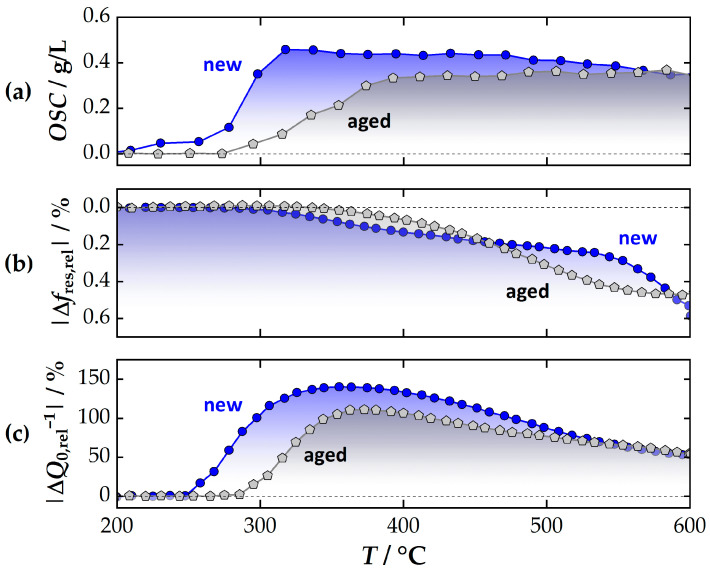
Experiment to evaluate catalyst aging: (**a**) *OSC* of the fresh and aged catalyst, (**b**) (relative) signal amplitude of the resonant frequency Δ*f*_res,rel_, and (**c**) the (inverse) quality factor Δ*Q*_0,rel_^−1^. Adapted original data from [35].

In the previous study, the influence of catalyst aging on the RF signals was also investigated using smaller catalyst cores (Setup B) [35]. For this purpose, fresh and aged TWCs of the same type were investigated. Hydrothermal aging was performed at 890 °C for 150 h. For the investigation, the resonant signals of both TWCs were recorded during heating to 600 °C at approx. 3 K/min. In parallel, rich and lean atmospheres were alternated to determine the RF response to the oxygen storage level. The exact conditions are described in [35]. In order to further investigate the differences between *f*_res_ and *Q*_0_^−1^, the temperature-dependent *OSC* of the catalyst was determined, and the associated (relative) signal amplitudes of the resonant Δ*f*_res,rel_ and Δ*Q*_0,rel_^−1^ were determined, referencing again the fully oxidized catalyst. The results are shown in Figure 7.

As Figure 7a demonstrates, the oxygen storage of the fresh catalyst is inactive up to about 250 °C. At higher temperatures, the *OSC* increases rapidly and remains approximately constant above 350 °C. In comparison, the aged TWC requires more than 300 °C for a measurable activation of the oxygen storage and reaches its maximum at around 400 °C. These observations are typical for aged catalysts whose washcoat surface area and catalytic activity have been reduced by a high-temperature hydrothermal treatment (“aging”) [15,67,68,69]. The differences between the fresh and aged catalysts are smaller at higher temperatures, which is most likely due to the fact that oxygen transport is also activated in the deeper layers of CZO. As a consequence, the *OSC* is more limited to the amount of oxygen storage material, rather than its surface area and catalytic activation. Comparing the temperature-dependent increase in *OSC* with the changes in the quality factor (Figure 7c), a good correlation between both parameters is found in the experiment. The amplitude of the quality factor increases significantly with the moment of oxygen storage activation and reaches a maximum above 350 °C. The difference in temperature required between fresh and aged catalysts is clearly determined with *Q*_0_^−1^ (≈50 °C). At higher temperatures, the two signals gradually converge. The general lower signal amplitudes with higher temperatures are again due to the increase in losses in the overall system. Nevertheless, the inverse quality factor is therefore suitable for both assessing aging (temperature shift) and diagnosing the remaining oxygen storage capacity (amplitude) at lower temperatures.

The direct comparison here illustrates the clear disadvantage of the resonant frequency as a feature for detecting aging (Figure 7b): The amplitude of *f*_res_ is hardly measurable, especially at low temperatures. A clear determination of the onset of the oxygen storage ability of CZO is almost impossible. Similarly, no clear correlation could be observed between the maximum amplitude and the *OSC*. The fact that *f*_res_ does not provide sufficient information under these conditions has also been observed to some extent in [25] (although this point was not directly addressed there). Instead, a different method was suggested to evaluate the aging state of the TWC using *f*_res_, which is based on the amount of water absorbed from the engine cold start (<150 °C). However, this method must be viewed critically from an application perspective, as the amount of water adsorbed at low temperatures is highly dependent on the current engine operation point, the operating history, and the ambient conditions (humidity of the air). Additionally, this method of detecting aging certainly works at least as well with the inverse quality factor. Overall, a method to directly measure the activation of the oxygen storage component (accompanied by the ‘light-off’ of TWC) via the inverse quality factor should offer noticeable advantages during cold start phases due to the higher and more resilient signal amplitudes and provide more reliable information about the TWC aging state.

### 3.4. Assessment for the RF-Based State Diagnosis of Three-Way Catalysts

**Figure 8:**
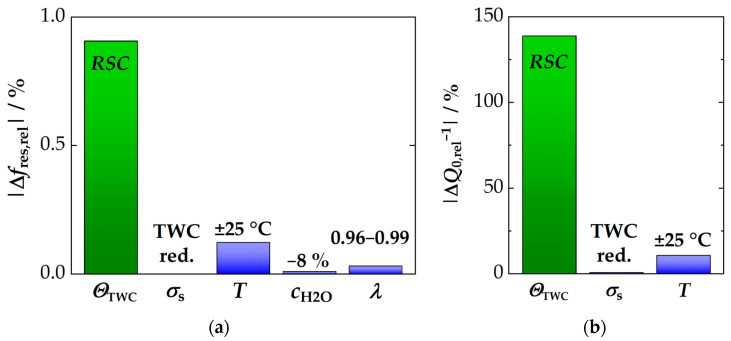
Comparison of various influences on the RF signals at 500 °C for (**a**) the resonant frequency and (**b**) the inverse quality factor: the signals to the full oxygen storage capacity (*RSC*, green) are compared with their standard deviations *σ*_s_ (of the fully reduced TWC) and with the impact of a temperature deviation of ±25 °C, the change in H_2_O concentration by 8% and change in oxygen stoichiometry under rich conditions (0.96 ≤ λ ≤ 0.99) (all blue).

As a final section of this study, the various influences on the RF system are now compared with the reaction to the oxygen storage to clarify and understand which factors are most important for operation. Such a comparison at 500 °C is shown in Figure 8, where Figure 8a shows the data for *f*_res_ and Figure 8b for *Q*_0_^−1^. The RF signal amplitudes to the full *RSC* of the TWC are shown in green in both figures. All secondary influences and interferences are then shown in blue. Firstly, each RF signal is compared to its standard deviation *σ*_s_ in case of a fully reduced TWC, as this is the worst-case scenario (for details, see Section 3.1). In addition, the impact of deviations in the estimated/predicted catalyst temperature (±25 °C) is shown (data adapted from [35]). Based on experiments in this study, the effect of an 8% change in water concentration is also demonstrated. More precisely, only the reduction of *c*_H_2_O_ from 10 to 2% was investigated. However, as shown in Figure 6b, the effect is approximately linear. Thus, certain generalizations can be made for typical operation conditions. Last, the influence of the exhaust stoichiometry under rich conditions is also included (range of 0.96 ≤ λ ≤ 0.99).

From the overall picture of the various influences some conclusions can be drawn: First, at 500 °C, the signal to the oxygen storage level of the TWC clearly predominates in both resonant frequency and inverse quality factor. The majority of the measurement signal, therefore, contains information on the CZO oxidation state. Second, the most important source of interference for the TWC state diagnosis arises from discrepancies in catalyst temperature. Therefore, it is particularly important to provide an accurate prediction of catalyst temperature during operation. Other influences only play a minor role at 500 °C. And third, the interfering effects have less impact on the quality factor signal as a whole. In particular, water concentration and oxygen stoichiometry do not contribute at all. It is also interesting to note that a lower *SNR* was found for the quality factor at 500 °C (see Figure 4). However, the effect of the temperature is assessed to be, by far, the more critical factor under these conditions. In fact, relative to the *RSC*-based signal amplitude, the temperature effect on *Q*_0_^−1^ is only half that of *f*_res_. In other words, although *Q*_0_^−1^ has a lower resolution, it responds more selectively to changes in the oxygen storage level. However, the advantages are expected to diminish with further increasing temperatures.

**Figure 9:**
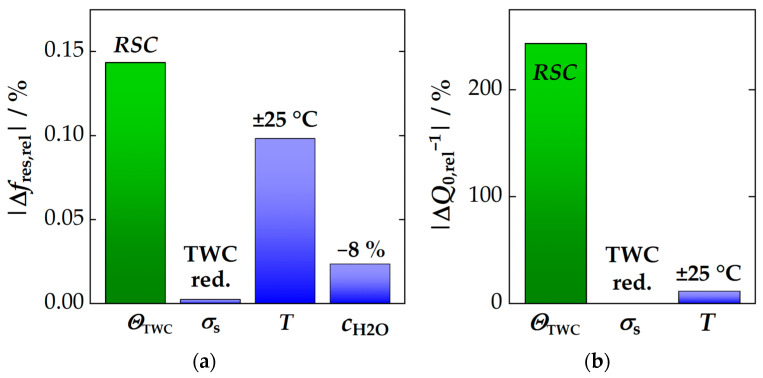
Comparison of various influences on the RF signals at 300 °C for (**a**) the resonant frequency and (**b**) the inverse quality factor: The signals to the full oxygen storage capacity (*RSC*, green) are compared with their standard deviations *σ*_s_ (of the fully reduced TWC) and with the effect of a temperature deviation of ±25 °C and the change in H_2_O concentration of 8%. The latter was derived by extrapolation of the data in Figure 6b.

Having considered a temperature at the upper end (500 °C), it would be beneficial to understand in the last step, if and how the impact of these interferences changes with lower temperatures. The results of the comparison at 300 °C, i.e., close to the catalyst light-off, are shown in Figure 9. Here, the disadvantages of the resonant frequency become even more obvious. Errors in the estimated catalyst temperature can have a huge impact on the interpretation of the current oxygen storage level *θ*_TWC_, as the amplitudes of both effects have equal dimensions (Figure 9a). Again, this is primarily attributed to the low signal amplitude of the resonant frequency under these conditions (see Figure 2a), which is due to both the low sensitivity of the *f*_res_ signal and the low *RSC* of the catalyst.

Furthermore, the (relative) impact of the water concentration on the resonant frequency also increases significantly at lower temperatures (see Figure 9a) The data shown here are based on the extrapolated data of Figure 6b (even if this effect was not measured quantitatively, this approach gives an impression of the expected extent of the interference at this conditions). In conclusion, evaluating the oxygen storage level by resonance frequency during engine operation at 300 °C is challenging due to both influences (temperature and water concentration).

Instead, the inverse quality factor shows a completely different picture (Figure 9b). The temperature cross effects at 300 °C are even lower than at 500 °C (≈ half the size—relative to *RSC* amplitude of *Q*_0_^−1^), which in the end originates from the high sensitivity of the *Q*_0_^−1^ signal and the rapidly activated SP hopping in CZO. Overall, the quality factor clearly represents the more reliable signal for RF-based state diagnosis at low temperatures.

Although the data on exhaust stoichiometry and the effect of water concentration in the exhaust have not been explicitly measured at 300 °C, it is clear from Figure 6 that the effect of water on the resonant frequency is also likely to increase at even lower temperatures. But, even in this regard, the inverse quality factor is expected to be less susceptible to errors. The most important challenge remains the accurate prediction of the catalyst temperature. Finally, we end this analysis with a summary of the key results.

## 4. Conclusions

In this study, various factors affecting the RF-based state diagnosis were investigated and weighted related to their impact on TWC operation. First, the signal response to the oxygen storage level was analyzed. In this context, the resonant frequency *f*_res_ and the (inverse) quality factor *Q*_0_^−1^ signals were linked to the fundamental measuring effects and correlated with the defect chemical mechanisms in the CZO-based oxygen storage component. In addition, the effects of various interferences were quantified. Finally, helpful information was provided to understand the microwave signal under typical operation conditions. Additionally, recommendations could be given for the application range of both measurement signals.

At low temperatures (near catalyst light-off), it is more advantageous to use the massive increase in conductivity in reduced CZO via the quality factor to reliably determine the oxygen storage level of the TWC. Under these conditions, the inverse quality factor is particularly sensitive to changes in oxygen storage level *θ*_TWC_ and offers high resolution with low susceptibility to interferences. This is a noteworthy result, as reducing emissions at low temperatures is still a key challenge in modern gasoline exhaust aftertreatment [10,11]. A control strategy using the quality factor signal could therefore help to further reduce cold-start emissions.

The advantages of the quality factor (apart from resilience to interference) are increasingly lost at higher temperatures, primarily due to the high losses in the cavity resonator. At the same time, the signal of the resonant frequency becomes more favorable, as both vibrational modes and the oxygen storage capacity are activated by temperature. At high temperatures, especially (above 500 °C), using the polarization mechanisms in CZO with the *f*_res_ signal to determine the oxygen storage level is more recommended.

This work has also shown that the major noise factor is associated with the assessment of the catalyst operation temperature. However, this problem can be minimized when both quality factors are used in combination within their recommended temperature zones. In particular, the signal amplitude of the inverse quality factor at low temperatures (<400 °C) is hardly affected by temperature. The impact of temperature on *f*_res_ is significantly greater at low temperatures, but plays only a minor role with increasing temperature due to the activation of the measurement effect. Finally, it was shown that the inverse quality factor also provides an elegant method for directly determining the aging state of the TWC during the heating phase. Both higher oxygen storage activation temperatures and lower oxygen storage capacities *OSC* can be diagnosed.

Overall, the data from this study indicate that the RF-based state diagnosis can provide profound information about the oxygen storage level in the entire temperature field of a TWC. The RF system is also robust against many interferences when properly configured. By applying the method to vehicles or as a research tool for improving TWC control strategies, the method can help to further reduce emissions from gasoline vehicles in the future.

## Figures and Tables

**Table 1:** Exhaust gas compositions with different oxygen stoichiometry *λ*. Additionally: 10% H_2_O and 10% CO_2_ in N_2_ *.

λ	Gas	c_Gas_	λ	Gas	c_Gas_
1.05	O_2_	1.42%	0.95	O_2_	0.25%
	CO	2000 ppm		CO	17,900 ppm
	NO	1000 ppm		NO	1000 ppm
	H_2_	650 ppm		H_2_	6000 ppm
1.04	O_2_	1.20%	0.96	O_2_	0.25%
	CO	2000 ppm		CO	14,600 ppm
	NO	1000 ppm		NO	1000 ppm
	H_2_	650 ppm		H_2_	4850 ppm
1.03	O_2_	0.95%	0.97	O_2_	0.25%
	CO	2000 ppm		CO	11,200 ppm
	NO	1000 ppm		NO	1000 ppm
	H_2_	650 ppm		H_2_	3750 ppm
1.02	O_2_	0.67%	0.98	O_2_	0.25%
	CO	2000 ppm		CO	8300 ppm
	NO	1000 ppm		NO	1000 ppm
	H_2_	650 ppm		H_2_	2600 ppm
1.01	O_2_	0.42%	0.99	O_2_	0.25%
	CO	2000 ppm		CO	5250 ppm
	NO	1000 ppm		NO	1000 ppm
	H_2_	650 ppm		H_2_	1750 ppm

* Total gas flow: 20 l/min, *GHSV* = 1.300 h^–1^.

## Data Availability

All relevant data presented in this article are stored according to institutional requirements and as such are not available online. However, all data used in this paper can be made available upon request to the authors.
